# Relation of raw and cooked vegetable consumption to blood pressure: the INTERMAP Study

**DOI:** 10.1038/jhh.2013.115

**Published:** 2013-11-21

**Authors:** Q Chan, J Stamler, I J Brown, M L Daviglus, L Van Horn, A R Dyer, L M Oude Griep, K Miura, H Ueshima, L Zhao, J K Nicholson, E Holmes, P Elliott

**Affiliations:** 1 Department of Epidemiology and Biostatistics, MRC-HPA Centre for Environment and Health, School of Public Health, Faculty of Medicine, Imperial College London, London, UK; 2 Department of Preventive Medicine, Feinberg School of Medicine, Northwestern University, Chicago, IL, USA; 3 Department of Health Science, Shiga University of Medical Science, Otsu, Japan; 4 Department of Epidemiology, Fu Wai Hospital and Cardiovascular Institute, Chinese Academy of Medical Sciences, Beijing, People’s Republic of China; 5 Section of Computational and Systems Medicine, Department of Surgery and Cancer, Faculty of Medicine, Imperial College London, London, UK

**Keywords:** cooked vegetables, raw vegetables, blood pressure, population study

## Abstract

Inverse associations have been reported of overall vegetable intake to blood pressure (BP); whether such relations prevail for both raw and cooked vegetables has not been examined. Here we report cross-sectional associations of vegetable intakes with BP for 2195 Americans ages 40–59 in the International Study of Macro/Micronutrients and Blood Pressure (INTERMAP) using four standardized multi-pass 24-h dietary recalls and eight BP measurements. Relations to BP of raw and cooked vegetables consumption, and main individual constituents were assessed by multiple linear regression. Intakes of both total raw and total cooked vegetables considered separately were inversely related to BP in multivariate-adjusted models. Estimated average systolic BP differences associated with two s.d. differences in raw vegetable intake (68 g per 1000 kcal) and cooked vegetable intake (92 g per 1000 kcal) were −1.9 mm Hg (95% confidence interval (CI): −3.1, −0.8; *P*=0.001) and −1.3 mm Hg (95% CI: −2.5, −0.2; *P*=0.03) without body mass index (BMI) in the full model; −1.3 mm Hg (95% CI: −2.4, −0.2; *P*=0.02) and −0.9 mm Hg (95% CI: −2.0, 0.2; *P*=0.1) with additional adjustment for BMI. Among commonly consumed individual raw vegetables, tomatoes, carrots, and scallions related significantly inversely to BP. Among commonly eaten cooked vegetables, tomatoes, peas, celery, and scallions related significantly inversely to BP.

## Introduction

Adverse blood pressure (BP), pre-hypertensive and hypertensive, is a key independent risk factor for major cardiovascular diseases, afflicting a high proportion of the adult population worldwide.^1, 2^ Approximately 25% of US adults have hypertension, defined as systolic blood pressure 140 mm Hg or higher and/or diastolic blood pressure 90 mm Hg or higher and/or current use of antihypertensive medication.^3^ Overall, an estimated 26% of the world’s adult population were hypertensive in 2000 and 29% are projected to have hypertension by 2025.^4^ Epidemiologic studies, cross-sectional and prospective, have reported lower average BP levels for vegetarians compared with nonvegetarians,^5, 6, 7, 8, 9^ and inverse relations of vegetable intake to BP.^10, 11^ Most of these studies reported relationship to BP of combined consumption of fruits and vegetables. The population-based cross-sectional International Study on Macro/Micronutrients and Blood Pressure (INTERMAP) found a significant inverse relation of vegetable protein intake to BP;^12^ dietary glutamic acid—the most common dietary amino acid, especially in vegetable protein—also related inversely to BP.^13^ No significant relation to BP of raw fruit and fruit juice intakes was observed in INTERMAP.^14^ Processing of vegetables influences their chemical composition and nutritional value,^15, 16^ for example, raw green leafy vegetables have significantly higher levels of antioxidants than cooked;^17^ the bioavailability of carotenoids from cooked tomatoes is higher compared with raw.^18, 19^ To the best of our knowledge, no data are available comparing associations of raw and cooked vegetable intakes with BP, and intakes of only a few individual vegetables (raw or cooked) have been related to BP.^20, 21, 22, 23^ We assessed these relationships using cross-sectional data on American participants in INTERMAP.

## Materials and Methods

### Population samples, field methods (1996–1999)

INTERMAP surveyed 4680 men and women ages 40–59 from Japan, People’s Republic of China, United Kingdom, and the United States (US).^24^ Here, we report data on the 2195 men and women from the eight US population samples. Participants were selected randomly from general and occupational populations. Each participant attended clinics four times, visits one and two on consecutive days, visits three and four on consecutive days on average 3 weeks later. Systolic and diastolic BP (first and fifth Korotkoff sounds) were measured twice at each visit by trained staff using a random zero sphygmomanometer (Hawksley, Lancing, UK), participants with bladder emptied, and seated for at least 5 min in a quiet room. Measurements of height and weight, and questionnaire data were obtained on demographic and other possible confounders, including education, occupation, physical activity, cigarette smoking, history of cardiovascular diseases, or diabetes mellitus, current use of a special diet, and use of antihypertensive and lipid-lowering drugs. Dietary data were collected at each visit by trained dietary interviewers, using the standardized in-depth multi-pass 24-h recall method.^25^ Food intakes were converted into nutrients and validated by the Nutrition Coordinating Center, University of Minnesota (Nutrition Data System for Research, version 2.9).^25, 26^ Daily alcohol consumption over the previous 7 days was obtained by interview twice. Each participant provided two 24-h urine collections, start and end timed at the research center; measurements included urinary volume, sodium (Na), potassium (K), calcium (Ca), and magnesium (Mg).^27^ Urinary measurements were used to assess validity of dietary recalls; gender-sample adjusted correlation coefficients between urinary and dietary Na, and urinary and dietary K were 0.46 and 0.58, respectively.^25^ The study received institutional ethics committee approval for each site; all participants gave written informed consent.

### Statistical methods

For vegetables, intake was calculated as grams per day and as percent total energy. Other food and nutrient intakes were similarly quantified, without and with inclusion of nutrients derived from dietary supplements. Cooked white potatoes and sweet potatoes were not included here because of their differences in composition (that is, high starch content) and nutritional values compared with other vegetables. For each person, measurements of BP and nutrients were averaged across the four visits; for urinary excretion measurements, across the two collections. For descriptive statistics, means and s.d., numbers and percentages were calculated by gender. Reliability of BP and vegetable intakes (mean of four visits) was estimated from the formula 1/(1+(ratio/2)) × 100, where the ratio is intra-individual variance/inter-individual variance. It was calculated from means of the first and second two visits to account for higher correlation between values on consecutive days. This gives a first approximation of effect of random error (day-to-day variability) on size of vegetable intake associations with BP; the statistic is estimated size of an observed coefficient as percent of theoretical coefficient in univariate regression analysis.^28, 29^ Adjusted mean and s.e. values of nutrient intake over four visits were used. Associations among nutrients/foods were explored by partial correlation, adjusted for sample, age, and gender. Multiple regression analyses were used to examine vegetable–BP relationships. Adjustment for confounders was done sequentially without and with BMI. Regression models were fit by sample; interactions were assessed for age and gender; departures from linearity tested with quadratic terms. Regression coefficients were expressed as mm Hg for two s.d. differences in vegetable intake. Two-tailed probability values <0.05 were considered statistically significant. Sensitivity analyses were also done, including adjustment also for total energy; inclusion only of nonhypertensive persons—those not on antihypertensive treatment and with SBP/DBP less than 140/90 mm Hg;^30^ a ‘nonintervened’ subgroup of people—individuals not on a special diet, not consuming nutritional supplements, not with diagnosed cardiovascular diseases/diabetes mellitus, and not reporting medication use for high BP/cardiovascular diseases/diabetes mellitus; exclusion of people with pre-defined high day-to-day variability of nutrient intakes or BP. Adjusted mean SBP and DBP by quartiles of raw vegetable intake were calculated by analysis of variance and plotted.

Analyses were with SAS version 9.3 (SAS Institute, Cary, NC, USA).

## Results

### Descriptive statistics, US INTERMAP participants

For the 2195 INTERMAP US participants, average unadjusted intakes of raw vegetables was 27.4 g per 1000 kcal for men and 36.5 g per 1000 kcal for women; of cooked vegetables (excluding white potatoes and sweet potatoes), 56.2 g per 1000 kcal for men and 65.2 g per 1000 kcal for women (Supplementary Table S1). Higher vegetable consumers tended to be older, and more educated, were less likely to smoke, had lower total energy intake, and lower BP and BMI than those with lower vegetable intakes. They also had a diet with higher intakes of raw fruits, low fat and fat-free dairy products, fiber-rich cereals and grains, fish and shellfish, and lower intakes of meats compared with lower vegetable consumers.

Commonly consumed raw vegetables were: cabbage, carrot, celery, cucumber, garlic, ginger, green pepper, lettuce (head, romaine), onions, scallions, and tomatoes (these raw vegetables accounted for 45% of total raw vegetable intake); cooked: broccoli, carrots, celery, green beans, green peppers, mushrooms, onions, green peas, scallions, tomatoes, canned tomatoes, and tomato sauces (these accounted for 39% of total cooked vegetable intake) (Supplementary Table S2).

Univariate estimates of reliability of vegetable intake, based on mean values from the four 24-h recalls/participant, ranged from about 39–54% of the theoretical coefficient (g per 1000 kcal) (Supplementary Table S3), similar for men and women. This implies that true associations with other variables may be larger than observed associations, for example, 1.85 (1/0.54) times larger for raw vegetables in the US population. BP reliability estimates were 91% (SBP) and 90% (DBP).

### Relation of vegetable intake to dietary nutrient composition

Among US participants, 157 individuals in the bottom quartile of both raw and cooked vegetable intake consumed an average of 21.5 g per 1000 kcal of vegetables. For 184 men and women in the top quartile of both raw and cooked vegetable intake, vegetable consumption was 202.3 g per 1000 kcal (Table 1). Individuals with higher consumption of both raw and cooked vegetables had higher intakes of fiber, starch, vegetable protein, glutamic acid, omega-3 polyunsaturated fatty acids, phosphorus (P), Mg, Ca, total iron (Fe), non-heme Fe, copper, vitamins A, C, E, B6, folacin, pantothenic acid, and they had lower intake levels of energy, sugar, saturated fatty acids, monounsaturated fatty acids, and trans fatty acids compared with persons with lower raw and cooked vegetable intakes (*P*<0.001). Higher raw and cooked vegetable consumers had significantly greater intakes of raw fruits, total fruits, and fiber-rich cereals and grains than lower vegetable consumers (*P*<0.001). Higher raw and cooked vegetable consumers had higher excretions of urinary K and Mg and a lower urinary Na to K ratio compared with those with lower raw and cooked vegetable intakes (*P*<0.001).

### Correlations between vegetable intakes and nutrients

Expressed as grams per 1000 kcal, partial correlation (*r*, adjusted for age, sex, and sample) for raw and cooked vegetables with each other was low order (*r*=0.10). Overall, correlations with multiple nutrients derived from vegetables were generally lower for raw vegetables (for example, *r*=0.32 with fiber) compared with those for cooked vegetables (for example, *r*=0.46 with fiber) (Supplementary Table S4). Partial correlations were generally low order for individual raw and cooked vegetables with each other (for example, the larger of these *r* values, raw lettuce with raw tomatoes, *r*=0.28; cooked onions with cooked green peppers, *r*=0.28; cooked onions with canned tomatoes, *r*=0.28) (Supplementary Table S5).

### Vegetable intakes and BP, multiple regression analyses

Findings on vegetable–BP relations were similar in analyses without and with inclusion of data on dietary supplement intakes; the former are tabulated here.

#### Raw vegetables

**Figure 1:**
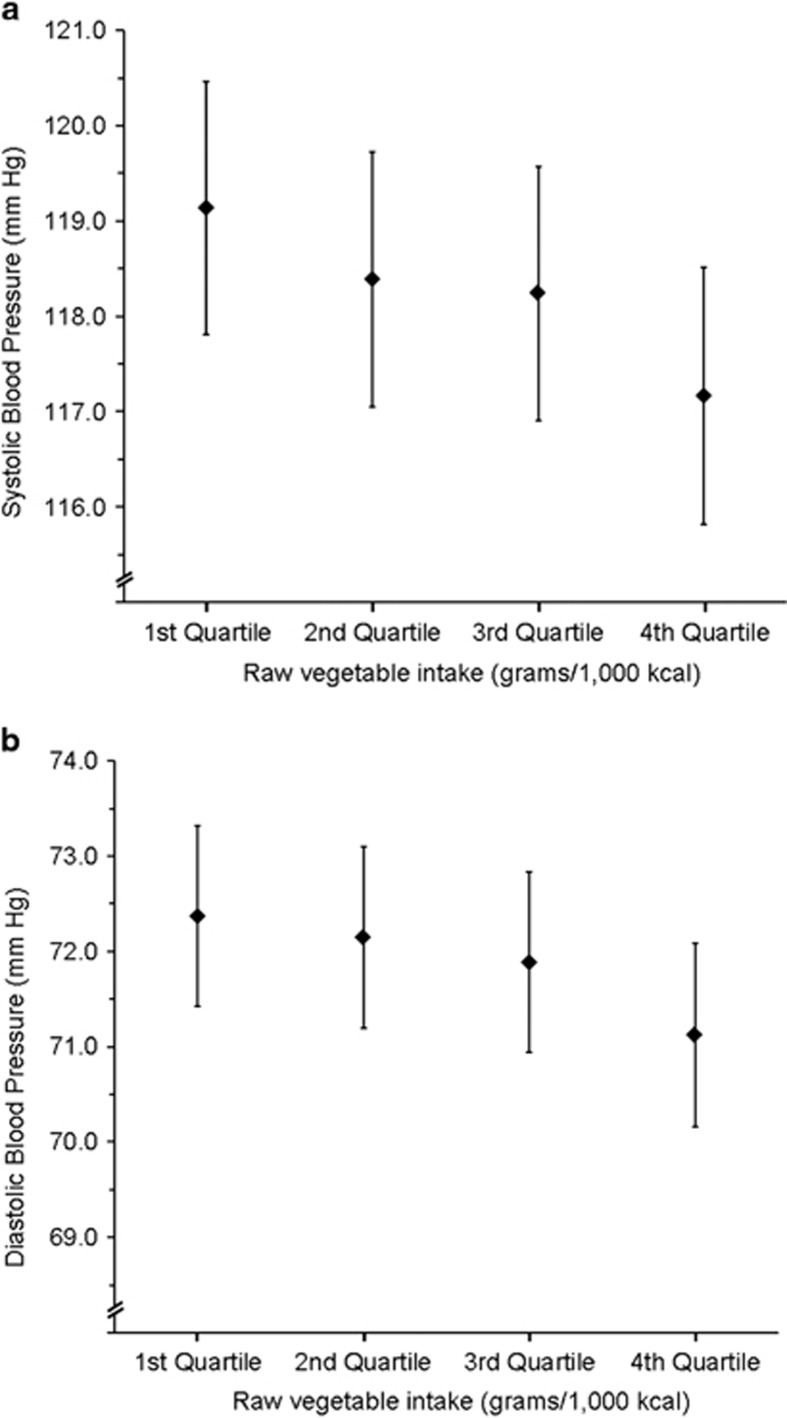
Mean (**a**) systolic and (**b**) diastolic BP (mm Hg) by quartiles of raw vegetable intake (g per 1000 kcal),^1^ adjusted for Model 3 covariates for 2195 US participants. Whiskers are 95% confidence intervals. *P* for trend for (**a**) *P*=0.02 and for (**b**) *P*=0.003.^1^ Quartile cut-offs for raw vegetable intake (g per 1000 kcal) were 7.6 (25th percentile), 20.1 (50th percentile), 37.1 (75th percentile) for men and 11.0, 27.7, 48.1 for women.^2^ Model 3: adjusted for age, gender, sample, education, physical activity, smoking status, history of cardiovascular disease or diabetes mellitus, family history of high BP, use of special diet, use of dietary supplement, urinary sodium, alcohol, polyunsaturated fatty acids, saturated fatty acids, and cholesterol.

There was an inverse relationship between raw vegetable intake and BP (Table 2). With raw vegetable intake higher by 2 s.d. (67.9 g per 1000 kcal), average SBP was lower by 1.9 mm Hg (95% CI −3.1, −0.8; *P*=0.001) without BMI in the multivariate controlled model 3, lower by 1.3 mm Hg (95% CI −2.4, −0.2; *P*=0.02) with BMI also in the model. Multivariate-adjusted mean values of BP by quartiles of raw vegetable intake showed a linear inverse relation (*P* for trend=0.02 for SBP and *P*=0.003 for DBP) (Figure 1). Sensitivity analyses yielded results qualitatively similar to the foregoing (Supplementary Table S6). BP differences were larger with exclusion of persons with high day-to-day variability in nutrient intake and/or BP. Among 12 commonly eaten raw vegetables, tomatoes, carrots, and scallions considered individually related significantly inversely to either SBP or DBP (Table 2). Tests for age/gender interaction and quadratic nonlinearity yielded nonsignificant results (Supplementary Table S7).

#### Cooked vegetables

In all multivariate regression analyses, relation to BP was inverse for total cooked vegetable intake (Table 2). With cooked vegetable intake higher by two s.d., average SBP was significantly lower by 1.3 mm Hg without BMI in the model, lower by 0.9 mm Hg with BMI also in the model (Model 3). Considered singly, four of 12 commonly eaten cooked vegetables—celery, peas, scallions, and tomatoes—related significantly inversely to either SBP or DBP (Table 2). Relationships between cooked vegetable intake and BP tended to be nonsignificantly inverse in sensitivity analyses (Supplementary Table S6). Tests for age–gender interaction and quadratic nonlinearity consistently yielded nonsignificant results (Supplementary Table S7). With raw vegetables and cooked vegetables considered together in the multivariate controlled model 3, the inverse raw vegetable–BP relation (*P*=0.02) was stronger than that of cooked vegetable with BP (Table 3).

#### Total vegetables

Multivariate regression analyses on the relationship between combined intake of raw and cooked vegetables and BP yielded significant findings (Table 3). With total vegetable intake higher by two s.d. (121.0 g per 1000 kcal), in multivariate controlled model 3, average SBP was lower by 2.2 mm Hg (95% CI −3.4, −1.01; *P*<0.001) without BMI, and lower by 1.5 mm Hg (95% CI −2.6, −0.4; *P*=0.009) with BMI also in the model. Relationships between total vegetable intake and BP in sensitivity analyses showed similar results (Supplementary Table S8).

## Discussion

Main finding here was a consistent multivariable controlled inverse relation of both raw and cooked vegetable intake to BP. The inverse raw vegetable–BP relation was stronger than that of cooked vegetables with BP. Among 12 commonly consumed raw vegetables, tomatoes, carrots, and scallions related significantly inversely to BP. Four of 12 commonly consumed individual cooked vegetables—tomatoes, peas, celery, and scallions—related significantly inversely to BP.

An observational study reported in 1985 on 32 individuals followed for 6 months on diets containing at least 40% uncooked foods (vegetables, seeds, nuts, fruits, and certified raw milk); intakes were significantly associated with lower DBP.^31^ BP of participants increased to previous levels when switched from high raw food diet to cooked diet (without altering caloric or Na intake). A study of 11 000 British men and women reported that daily consumption of raw salad was associated with significantly lower mortality from ischemic heart disease.^32^ A recent cohort study of 20 000 men and women in the Netherlands, using food frequency questionnaire data on 7 raw vegetables and 13 cooked vegetables, reported that raw vegetable intake was significantly inversely associated with ischemic stroke;^33^ raw fruit and vegetable consumption was also inversely related to coronary heart disease.^34^

Earlier studies of vegetarians and meat eaters showed that BP levels were lower in individuals self-reporting vegetarian diets compared with nonvegetarians.^6, 7^ Fruit and vegetable consumption has been associated with decreased BP in randomized controlled trials, particularly in hypertensive persons.^8, 9^ Lower BP with a high-vegetable diet may hypothetically be due to higher dietary fiber,^35^ vegetable protein,^12^ glutamic acid (predominant amino acid in vegetable protein),^13^ vitamins (for example, vitamin A, C, E),^36^ and minerals (for example, P, Ca, Mg).^37, 38^ Mechanisms remain to be elucidated, and demonstration of the consistency of these associations is lacking.

Cooking vegetables changes their chemical composition, possibly with different effects of various cooking methods.^15, 16, 17^ Changes include lowering of antioxidant compounds (especially water-soluble and heat-sensitive nutrients, such as vitamin C, glucosinolate, and polyphenols) and their bioaccessibility.^15, 16, 17^ For such vegetables as cauliflower and broccoli, water-soluble glucosinolates are lost during boiling because of leaching into water, but with steaming the content of glucosinolates is retained.^15, 16^ Although consumption of unprocessed vegetables is advocated, some studies show that bioavailability of many protective compounds (particularly carotenoids) is enhanced when vegetables are cooked.^16, 19, 39^ Assessment of the comparative healthfulness of raw and cooked vegetables is complex; relationships between plant foods and human biological systems, and the possible mechanisms of these relationships remain largely unidentified. These new data on vegetables and BP indicate that higher intakes of both raw and cooked vegetables are aspects of an overall healthier and more nutritious diet. Thus, while etiologic conclusions on these relationships are presently premature, the multivariate controlled results support the concept that fare high in vegetables may reduce risk of adverse BP, and support recommendations for high population-wide intake of vegetables, raw and cooked.

Strengths of the INTERMAP findings reported here include their derivation from eight diverse US population samples totaling 2195 women and men; food intake data based on four in-depth multi-pass standardized 24-h dietary recalls per person; multivariate analyses controlling extensively for possible confounders (dietary and non-dietary). Limitations include lack of data on persons outside the age range 40–59 years, and outside the U.S. INTERMAP samples. The cross-sectional nature of these results is a further limitation, as is the possibility of regression dilution bias, systematic bias, reverse causation, and residual confounding.

In conclusion, the INTERMAP population study has found consistent inverse relations of both total raw and total cooked vegetable intakes to BP. It also found an independent significant cross-sectional relation to BP of several individual vegetables, raw (carrots, tomatoes, scallions) and cooked (celery, peas, scallions, tomatoes). Further studies, observational (especially prospective) and interventional (randomized controlled trials), are required to clarify the issues of mechanism and causation.


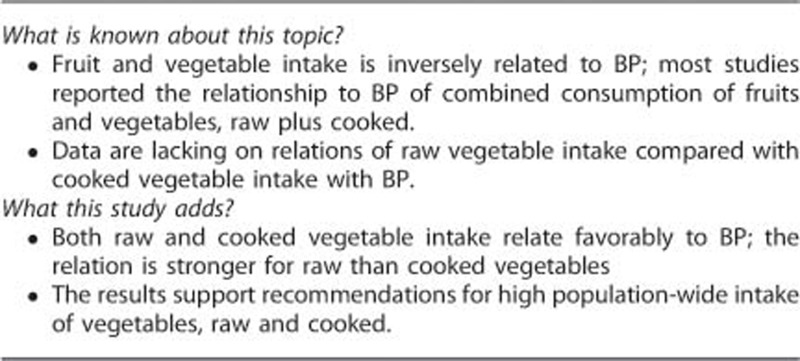


## Figures and Tables

**Table 1:** Age-sex-sample adjusted mean and standard error (s.e.), nutrient intakes in individuals with lower vegetable intake compared with individuals with higher vegetable intake, for US INTERMAP participants (*N*=335)

*Variable, units*	*157 individuals with low raw vegetable*^a^ *and low cooked vegetable*^b^ *intakes*	*184 individuals with high raw vegetable*^a^ *and high cooked vegetable*^b^ *intakes*	*T-Score*
	*Adjusted mean (s.e.)*	*Adjusted mean (s.e.)*	
Men, %	52.9	50.5	
Total vegetables, g per 1000 kcal	21.5 (4.1)	202.3 (3.8)	31.7
Energy, kcal per 24 h	2402.3 (48.8)	2055.5 (45.4)	−5.1
Fiber, g per 1000 kcal	6.7 (0.3)	12.3 (0.3)	13.5
Starch, %kcal	21.0 (0.5)	23.9 (0.5)	4.2
Sugar, %kcal	29.0 (0.7)	25.5 (0.6)	−3.7
Animal protein, %kcal	9.4 (0.3)	10.5 (0.3)	2.6
Vegetable protein, %kcal	4.3 (0.1)	6.3 (0.1)	9.9
Glutamic acid, %kcal	2.7 (0.05)	3.3 (0.04)	8.3
Total saturated fatty acids, %kcal	11.5 (0.3)	9.5 (0.2)	−5.6
Total monounsaturated fatty acids, %kcal	12.5 (0.2)	11.4 (0.2)	−3.2
Total PUFA, %kcal	6.7 (0.2)	7.2 (0.2)	2.0
Omega-3 PUFA, %kcal	0.7 (0.03)	0.9 (0.03)	5.2
Omega-6 PUFA, %kcal	6.1 (0.2)	6.4 (0.2)	1.6
Total trans fatty acids, %kcal	2.0 (0.1)	1.6 (0.1)	−5.2
Cholesterol, mg per 1000 kcal	134.5 (4.7)	123.7 (4.4)	−1.7
Phosphorus, mg per 1000 kcal	543.0 (11.1)	654.7 (10.3)	7.2
Magnesium, mg per 1000 kcal	127.0 (3.5)	183.0 (3.3)	11.4
Calcium, mg per 1000 kcal	333.9 (12.3)	402.0 (11.5)	4.0
Iron, mg per 1000 kcal	6.8 (0.2)	8.9 (0.2)	6.7
Heme iron, mg per 1000 kcal	0.5 (0.03)	0.5 (0.02)	0.5
Non-heme iron, mg per 1000 kcal	6.3 (0.2)	8.4 (0.2)	6.7
Copper, mg per 1000 kcal	0.6 (0.02)	0.8 (0.02)	9.4
Vitamin A, IU per 1000 kcal	2081.3 (343.9)	6948.4 (320.5)	10.1
Vitamin C, mg per 1000 kcal	37.8 (3.1)	82.2 (2.9)	10.3
Vitamin E, mg per 1000 kcal	4.1 (0.2)	5.1 (0.2)	4.2
Vitamin B6, mg per 1000 kcal	0.7 (0.02)	1.1 (0.02)	12.0
Thiamin, mg per 1000 kcal	0.8 (0.02)	0.9 (0.02)	6.6
Riboflavin, mg per 1000 kcal	0.9 (0.02)	1.0 (0.02)	2.8
Folacin, mcg per 1000 kcal	104.1 (4.4)	183.8 (4.1)	12.8
Pantothenic acid, mg per 1000 kcal	2.0 (0.1)	2.6 (0.1)	7.8
Raw fruits, g per 1000 kcal	37.7 (5.7)	78.9 (5.3)	5.2
Total fruits, g per 1000 kcal	88.6 (9.0)	139.0 (8.4)	4.0
Low fat dairy products, g per 1000 kcal	38.6 (7.3)	59.0 (6.8)	2.0
Fiber-rich cereals and grains, g per 1000 kcal	93.5 (3.9)	121.2 (3.6)	5.1
Red and processed meats, g per 1000 kcal	37.2 (2.0)	30.9 (1.9)	−2.2
Nuts and seeds, g per 1000 kcal	3.7 (0.6)	3.0 (0.5)	−0.9
Fish and shellfish, g per 1000 kcal	7.9 (1.3)	13.8 (1.2)	3.2
Alcohol, g per 24 h	8.9 (1.1)	6.4 (1.1)	1.6
Urinary sodium, mmol per 24 h	155.3 (5.1)	157.4 (4.7)	0.3
Urinary potassium, mmol per 24 h	49.3 (1.7)	67.0 (1.5)	7.7
Urinary sodium/potassium ratio	3.4 (0.1)	2.5 (0.1)	−6.3
Urinary calcium, mmol per 24 h	4.1 (0.2)	4.2 (0.2)	0.1
Urinary magnesium, mmol per 24 h	4.0 (0.1)	4.6 (0.1)	3.4

Abbreviation: PUFA, polyunsaturated fatty acids.

a Quartile cut-offs for raw vegetable intake (g per 1000 kcal) were 7.6 (25th percentile), 20.1 (50th percentile), 37.1 (75th percentile) for men and 11.0, 27.7, 48.1 for women.

b Quartile cut-offs for cooked vegetable intake (g per 1000 kcal) were 26.9 (25th percentile), 48.6 (50th percentile), 76.4 (75th percentile) for men and 30.9, 54.5, 86.0 for women.

**Table 2:** Estimated average differences and 95% confidence intervals in BP, intake of raw vegetables and cooked vegetables higher by two s.d., US INTERMAP participants (*N*=2195)

*All raw vegetables: 2 s.d.=67.9 g per 1000 kcal*								
*Model*	*Systolic BP*				*Diastolic BP*			
			*Adjusted for BMI*				*Adjusted for BMI*	
	*ΔBP (95% CI)*	P*-value*	*ΔBP (95% CI)*	P*-value*	*ΔBP (95% CI)*	P*-value*	*ΔBP (95% CI)*	P*-value*
1	−2.27 (−3.41,−1.12)	1.0 × 10^−4^	−1.61 (−2.69, −0.53)	0.003	−1.16 (−1.94, −0.39)	0.003	−0.81 (−1.56, −0.06)	0.033
2	−2.11 (−3.25, −0.98)	2.5 × 10^−4^	−1.44 (−2.53, −0.36)	0.009	−1.19 (−1.97, −0.42)	0.002	−0.81 (−1.56, −0.06)	0.034
3	−1.92 (−3.07, −0.77)	0.001	−1.32 (−2.43, −0.22)	0.019	−1.03 (−1.81, −0.24)	0.011	−0.69 (−1.45, 0.08)	0.079
4	−1.83 (−2.99, −0.68)	0.002	−1.25 (−2.36, −0.15)	0.027	−1.05 (−1.85, −0.26)	0.009	−0.72 (−1.49, 0.05)	0.066

*Individual raw vegetable intake* a *, g per 1000 kcal*								
*Model 3 for all participants*	*Systolic BP*				*Diastolic BP*			
			*Adjusted for BMI*				*Adjusted for BMI*	
	*ΔBP (95% CI)*	P*-value*	*ΔBP (95% CI)*	P*-value*	*ΔBP (95% CI)*	P*-value*	*ΔBP (95% CI)*	P*-value*
Raw carrot (2 s.d.=18.46)	−1.51 (−2.63, −0.40)	0.008	−1.29 (−2.36, −0.23)	0.017	−0.48 (−1.24, 0.29)	0.221	−0.35 (−1.09, 0.39)	0.354
Raw scallion (2 s.d.=2.72)	−1.14 (−2.25, −0.03)	0.043	−1.02 (−2.08, 0.04)	0.060	−0.37 (−1.12, 0.39)	0.344	−0.30 (−1.03, 0.44)	0.431
Raw tomato (2 s.d.=31.79)	−1.02 (−2.13, 0.09)	0.072	−0.68 (−1.74, 0.38)	0.207	−0.87 (−1.62, −0.11)	0.025	−0.67 (−1.41, 0.06)	0.072

*All cooked vegetables: 2 s.d. = 92.3 g per 1000 kcal*								
*Model*	*Systolic BP*				*Diastolic BP*			
			*Adjusted for BMI*				*Adjusted for BMI*	
	*ΔBP (95% CI)*	P*-value*	*ΔBP (95% CI)*	P*-value*	*ΔBP (95% CI)*	P*-value*	*ΔBP (95% CI)*	P*-value*
1	−1.62 (−2.76, −0.47)	0.006	−1.18 (−2.26, −0.09)	0.033	−0.62 (−1.40, 0.15)	0.115	−0.38 (−1.13, 0.36)	0.314
2	−1.56 (−2.69, −0.42)	0.007	−1.01 (−2.10, 0.07)	0.067	−0.60 (−1.37, 0.18)	0.132	−0.28 (−1.04, 0.47)	0.460
3	−1.31 (−2.47, −0.16)	0.026	−0.90 (−2.01, 0.20)	0.108	−0.40 (−1.19, 0.39)	0.315	−0.17 (−0.94, 0.60)	0.664
4	−1.29 (−2.45, −0.14)	0.028	−0.89 (−1.99, 0.21)	0.114	−0.41 (−1.20, 0.38)	0.311	−0.17 (−0.94, 0.60)	0.655

*Individual cooked vegetable intake* b *, g per 1000 kcal*								
*Model 3 for all participants*	*Systolic BP*				*Diastolic BP*			
			*Adjusted for BMI*				*Adjusted for BMI*	
	*ΔBP (95% CI)*	P*-value*	*ΔBP (95% CI)*	P*-value*	*ΔBP (95% CI)*	P*-value*	*ΔBP (95% CI)*	P*-value*
Cooked celery (2 s.d.=6.02)	−1.73 (−2.83, −0.62)	0.002	−1.47 (−2.52, −0.41)	0.006	−0.70 (−1.45, 0.06)	0.071	−0.55 (−1.28, 0.18)	0.141
Cooked pea (2 s.d.=9.26)	−0.45 (−1.56, 0.66)	0.427	−0.33 (−1.39, 0.73)	0.546	−1.03 (−1.79, −0.28)	0.008	−0.96 (−1.70, −0.23)	0.010
Cooked scallion (2 s.d.=3.24)	−1.23 (−2.35, −0.12)	0.030	−0.82 (−1.88, 0.25)	0.132	−0.83 (−1.59, −0.06)	0.033	−0.59 (−1.33, 0.15)	0.120
Cooked tomato (2 s.d.=14.60)	−1.13 (−2.23, −0.03)	0.044	−0.84 (−1.89, 0.22)	0.119	−0.54 (−1.30, 0.21)	0.156	−0.38 (−1.11, 0.35)	0.313

Abbreviations: BMI, body mass index; BP, blood pressure; CI, confidence interval; INTERMAP, International Study of Macro/Micronutrients and Blood Pressure.

Model 1: adjusted for age, gender, sample.

Model 2: adjusted for Model 1 variables plus education, physical activity, smoking status, history of cardiovascular disease or diabetes mellitus, family history of high BP, use of special diet, use of dietary supplement, urinary sodium, and alcohol.

Model 3: adjusted for Model 2 variables plus polyunsaturated fatty acids, saturated fatty acids, and cholesterol.

Model 4: adjusted for Model 3 variables plus total fruit.

a Other individual raw vegetables not statistically significantly related to BP.

b Other individual cooked vegetables not statistically significantly related to BP.

**Table 3:** Estimated average differences and 95% confidence intervals in BP, intake of raw vegetables and cooked vegetables higher by 2 s.d., US INTERMAP participants (*N*=2195)

	*Systolic BP*				*Diastolic BP*			
		*Adjusted for BMI*				*Adjusted for BMI*		
	Δ*BP (95% CI)*	P*-value*	*ΔBP (95% CI)*	P*-value*	*ΔBP (95% CI)*	P*-value*	*ΔBP (95% CI)*	P*-value*
*All raw vegetables and all cooked vegetables (g per 1000 kcal) in Model 3*								
Raw vegetables (2 s.d.=67.9)	−1.84 (−3.00, −0.69)	0.002	−1.28 (−2.38, −0.17)	0.023	−1.01 (−1.79, −0.22)	0.012	−0.68 (−1.45, 0.09)	0.083
Cooked vegetables (2 s.d.=92.3)	−1.20 (−2.36, −0.05)	0.041	−0.83 (−1.94, 0.27)	0.139	−0.34 (−1.14, 0.45)	0.393	−0.13 (−0.90, 0.64)	0.737
								
*Raw vegetables and cooked vegetables combined as total vegetables (g per 1000 kcal) in Model 3*								
Total vegetables (2 s.d.=121.0)	−2.19 (−3.38, −1.01)	2.8 × 10^−4^	−1.52 (−2.65, −0.38)	0.009	−0.93 (−1.74, −0.12)	0.024	−0.54 (−1.33, 0.24)	0.175

Abbreviations: BMI, body mass index; BP, blood pressure; CI, confidence interval.

Model 3: adjusted for age, gender, sample, education, physical activity, smoking status, history of cardiovascular disease or diabetes mellitus, family history of high BP, use of special diet, use of dietary supplement, urinary sodium, alcohol, polyunsaturated fatty acids, saturated fatty acids, and cholesterol.
