# Supplementary material for: Relation of raw and cooked vegetable consumption to blood pressure: the INTERMAP Study
Source: J Hum Hypertens. 2013 Nov 21;28(6):353–9. doi: 10.1038/jhh.2013.115 (PMC4013197; doi:10.1038/jhh.2013.115)
Supplement: Supplementary Information [file jhh2013115x1.doc]

*Supplementary materials for online publication*

**Relations of raw and cooked vegetable consumption to blood pressure:**

**The INTERMAP Study**

Queenie Chan, Jeremiah Stamler, Ian J. Brown, Martha L. Daviglus, Linda Van Horn, Alan R. Dyer, Linda M. Oude Griep, Katsuyuki Miura, Hirotsugu Ueshima, Liancheng Zhao, Jeremy K. Nicholson, Elaine Holmes and Paul Elliott for the INTERMAP Research Group

Table S1 Mean and standard deviation or frequency and percent, study variables by gender and total vegetable intake (g/1,000 kcal) for U.S. INTERMAP participants (N=2,195).

|  | Men (N=1,103) | | | | |  | Women (N=1,092) | | | | |
| --- | --- | --- | --- | --- | --- | --- | --- | --- | --- | --- | --- |
| Mean (SD) raw vegetable intake | 27.4 (29.0) g/1,000 kcal | | | | |  | 36.5 (37.8) g/1,000 kcal | | | | |
| Mean (SD) cooked vegetable intake | 56. 2 (41.7) g/1,000 kcal | | | | |  | 65.2 (49.8) g/1,000 kcal | | | | |
|  | Low  vegetable intake^1^ | | | High  vegetable intake^1^ | | | Low  vegetable intake^2^ | | | High  vegetable intake^2^ | |
| Variable, units | (n=551) | |  | (n=552) | |  | (n=546) | |  | (n=546) | |
|  | Mean | (SD) |  | Mean | (SD) |  | Mean | (SD) |  | Mean | (SD) |
| Age, years | 48.7 | (5.3) |  | 49.4 | (5.4) |  | 48.6 | (5.5) |  | 49.8 | (5.3) |
| Systolic blood pressure, mm Hg | 121.8 | (12.8) |  | 119.0 | (12.4) |  | 117.7 | (14.4) |  | 115.9 | (15.2) |
| Diastolic blood pressure, mm Hg | 76.0 | (10.1) |  | 75.4 | (9.1) |  | 71.5 | (9.2) |  | 70.7 | (9.2) |
| Body mass index, kg/m^2^ | 29.5 | (5.1) |  | 28.7 | (5.0) |  | 29.4 | (6.6) |  | 28.0 | (6.6) |
| Moderate and heavy physical activity, hours/24‑h | 3.9 | (3.5) |  | 3.1 | (3.1) |  | 3.1 | (3.0) |  | 2.9 | (2.9) |
| Education, years | 14.9 | (3.2) |  | 15.8 | (3.0) |  | 14.1 | (2.9) |  | 14.9 | (2.9) |
| Energy, kcal/24‑h | 2739.2 | (708.3) |  | 2478.8 | (655.6) |  | 1975.0 | (469.2) |  | 1776.7 | (457.5) |
| Raw vegetables, g/1,000 kcal | 14.7 | (12.9) |  | 40.0 | (34.6) |  | 19.4 | (15.9) |  | 53.5 | (45.0) |
| Cooked vegetables, g/1,000 kcal | 30.1 | (16.7) |  | 82.2 | (43.0) |  | 35.6 | (19.9) |  | 94.7 | (53.2) |
| Raw fruits, g/1,000 kcal | 33.2 | (46.3) |  | 53.7 | (67.6) |  | 48.3 | (64.8) |  | 71.4 | (73.0) |
| Total fruits, g/1,000 kcal | 82.9 | (91.1) |  | 111.9 | (109.1) |  | 97.0 | (101.7) |  | 132.1 | (103.9) |
| Low fat dairy products, g/1,000 kcal | 35.0 | (72.1) |  | 47.0 | (87.4) |  | 48.6 | (84.1) |  | 69.4 | (98.4) |
| Fiber-rich cereals and grains, g/1,000 kcal | 95.8 | (39.3) |  | 121.1 | (57.7) |  | 102.2 | (41.3) |  | 117.3 | (47.8) |
| Red and processed meats, g/1,000 kcal | 42.4 | (25.6) |  | 37.7 | (24.2) |  | 34.2 | (24.5) |  | 31.4 | (25.2) |
| Nuts and seeds, g/1,000 kcal | 3.1 | (5.2) |  | 3.1 | (5.3) |  | 3.3 | (6.0) |  | 2.6 | (4.6) |
| Fish and shellfish, g/1,000 kcal | 7.9 | (13.4) |  | 11.2 | (16.8) |  | 7.9 | (12.6) |  | 11.5 | (17.6) |
| 14-day alcohol, g/24‑h | 11.6 | (19.6) |  | 9.5 | (14.2) |  | 3.1 | (7.1) |  | 3.6 | (7.5) |
|  |  |  |  |  |  |  |  |  |  |  |  |
| Urinary sodium, mmol/24‑h | 179.2 | (63.5) |  | 186.1 | (61.2) |  | 142.1 | (46.9) |  | 142.4 | (49.6) |
| Urinary potassium, mmol/24‑h | 60.6 | (20.6) |  | 68.2 | (21.7) |  | 46.8 | (16.5) |  | 54.9 | (18.3) |
| Urinary sodium/potassium ratio | 3.2 | (1.2) |  | 3.0 | (1.1) |  | 3.3 | (1.4) |  | 2.8 | (1.1) |
| Urinary calcium, mmol/24-h | 4.6 | (2.5) |  | 4.5 | (2.1) |  | 3.7 | (2.0) |  | 4.0 | (2.0) |
| Urinary magnesium, mmol/24-h | 4.6 | (1.6) |  | 4.8 | (1.5) |  | 3.6 | (1.4) |  | 4.0 | (1.5) |
|  |  |  |  |  |  |  |  |  |  |  |  |
|  | N | (%) |  | N | (%) |  | N | (%) |  | N | (%) |
| Obese^3^ | 221 | (40.1%) |  | 117 | (21.2%) |  | 219 | (40.1%) |  | 158 | (28.9%) |
| Hypertensive^4^ | 161 | (29.2%) |  | 148 | (26.8%) |  | 145 | (26.6%) |  | 141 | (25.8%) |
| Current cigarette smokers | 141 | (25.6%) |  | 70 | (12.7%) |  | 109 | (20.0%) |  | 49 | (9.0%) |
| Current alcohol drinkers | 463 | (84.0%) |  | 436 | (79.0%) |  | 336 | (61.5%) |  | 358 | (65.6%) |
| Special diet: weight loss, weight gain, vegetarian, salt reduced, diabetic, fat modified or any other | 59 | (10.7%) |  | 97 | (17.6%) |  | 96 | (17.6%) |  | 149 | (27.3%) |
| Taking dietary supplements | 237 | (43.0%) |  | 281 | (50.9%) |  | 281 | (51.5%) |  | 337 | (61.7%) |
| Taking antihypertensive, other cardiovascular disease^6^ or diabetes medication | 156 | (28.3%) |  | 167 | (30.3%) |  | 159 | (29.1%) |  | 162 | (29.7%) |
| History of heart attack, other heart disease, stroke or diabetes | 96 | (17.4%) |  | 79 | (14.3%) |  | 94 | (17.2%) |  | 74 | (13.6%) |
| Family history of hypertension in any first degree relative |  |  |  |  |  |  |  |  |  |  |  |
| – Yes | 339 | (61.5%) |  | 349 | (63.2%) |  | 391 | (71.6%) |  | 412 | (75.5%) |
| – Unknown | 154 | (27.9%) |  | 138 | (25.0%) |  | 100 | (18.3%) |  | 97 | (17.8%) |

^1^ Median total vegetable intake = 72.5 g/1,000 kcal.

^2^ Median total vegetable intake = 85.8 g/1,000 kcal.

^3^ Body mass index ≥30.0 kg/m^2^.

^4^ Systolic blood pressure ≥140 mm Hg, or diastolic blood pressure ≥90 mm Hg, or taking antihypertensive medication for high blood pressure.

^5^ Includes lipid-lowering medication.

Table S2 Mean and standard deviation (SD), individual raw and cooked vegetable intake (g/24-h) of consumers, US INTERMAP participants (N=2,195).

| Individual vegetable |  | Amount (g/24-h) | |
| --- | --- | --- | --- |
|  | Number of consumers | Mean | (SD) |
| ***Raw vegetable:*** |  |  |  |
| Raw tomato | 1582 | 25.78 | (29.99) |
| Raw lettuce | 1543 | 22.46 | (23.22) |
| Raw onion | 1262 | 6.76 | (8.71) |
| Raw garlic | 1117 | 0.52 | (0.75) |
| Raw carrot | 996 | 13.49 | (22.97) |
| Raw celery | 700 | 5.44 | (7.89) |
| Raw romaine | 690 | 16.92 | (17.39) |
| Raw scallion | 644 | 3.42 | (4.11) |
| Raw cucumber | 506 | 13.63 | (18.42) |
| Raw green pepper | 473 | 4.03 | (4.77) |
| Raw ginger root | 391 | 0.50 | (1.88) |
| Raw cabbage | 384 | 10.13 | (11.43) |
|  |  |  |  |
| ***Cooked vegetable:*** |  |  |  |
| Cooked fresh or frozen onion | 1876 | 14.05 | (14.76) |
| Cooked fresh or frozen carrot | 1056 | 12.72 | (14.69) |
| Cooked fresh or frozen green pepper | 885 | 5.42 | (7.70) |
| Canned tomato sauce | 868 | 18.64 | (19.74) |
| Cooked fresh or frozen mushroom | 799 | 6.72 | (10.13) |
| Cooked fresh or frozen celery | 790 | 5.33 | (6.80) |
| Canned tomato | 691 | 33.24 | (28.58) |
| Cooked fresh or frozen tomato | 691 | 14.49 | (22.53) |
| Cooked green pea | 578 | 10.93 | (15.26) |
| Cooked fresh or frozen green bean | 581 | 17.02 | (20.24) |
| Cooked fresh or frozen broccoli | 528 | 26.91 | (26.25) |
| Cooked scallion | 509 | 4.63 | (5.89) |

Table S3 Ratio of within- to between-person variance^1^ and its effect on regression coefficients for raw vegetables, cooked vegetables, total vegetables and blood pressure, U.S. INTERMAP participants (N=2,195).

| Variable, units | Gender | Ratio of  within to between variance | Mean of First and Repeat visits: observed regression coefficient as % of theoretical coefficient |
| --- | --- | --- | --- |
| Raw vegetables, | Men (N=1,103) | 2.97 | 40.3 |
| g/1,000kcal | Women (N=1,092) | 1.71 | 53.9 |
|  | All (N=2,195) | 2.34 | 47.1 |
|  |  |  |  |
| Cooked vegetables, | Men (N=1,103) | 2.19 | 47.8 |
| g/1,000kcal | Women (N=1,092) | 3.13 | 39.0 |
|  | All (N=2,195) | 2.65 | 43.4 |
|  |  |  |  |
| Total vegetables, | Men (N=1,103) | 1.73 | 53.6 |
| g/1,000kcal | Women (N=1,092) | 1.75 | 53.3 |
|  | All (N=2,195) | 1.74 | 53.4 |
|  |  |  |  |
| Systolic blood pressure | Men (N=1,103) | 0.21 | 90.3 |
| mm Hg | Women (N=1,092) | 0.17 | 92.1 |
|  | All (N=2,195) | 0.19 | 91.2 |
|  |  |  |  |
| Diastolic blood pressure | Men (N=1,103) | 0.22 | 90.1 |
| mm Hg | Women (N=1,092) | 0.22 | 90.1 |
|  | All (N=2,195) | 0.22 | 90.1 |

^1^ Ratio estimated separately for gender subgroups.

Table S4 Gender-age-sample adjusted Pearson correlation between raw vegetables, cooked vegetables, and nutrients, intake per 1,000 kcal (N=2,195).

| Variable, units | Raw vegetables, g/1,000 kcal | Cooked vegetables, g/1,000 kcal |
| --- | --- | --- |
| Cooked vegetables, g/1,000 kcal | 0.10 |  |
| Fiber, g/1,000 kcal | 0.32 | 0.46 |
| Starch, %kcal | 0.01 | 0.16 |
| Total protein, %kcal | 0.10 | 0.26 |
| Animal protein, %kcal | 0.02 | 0.08 |
| Vegetable protein, %kcal | 0.18 | 0.36 |
| Glutamic acid, %kcal | 0.11 | 0.26 |
| Total SFA, %kcal | -0.13 | -0.21 |
| Total MFA, %kcal | -0.06 | -0.18 |
| Total PFA, %kcal | 0.13 | -0.08 |
| Omega-3 PFA, %kcal | 0.23 | 0.05 |
| Omega-6 PFA, %kcal | 0.12 | -0.09 |
| Total TFA, %kcal | -0.12 | -0.17 |
| Cholesterol, mg/1,000kcal | -0.05 | -0.06 |
| Phosphorus, mg/1,000 kcal | 0.14 | 0.23 |
| Magnesium, mg/1,000 kcal | 0.28 | 0.37 |
| Calcium, mg/1,000 kcal | 0.09 | 0.14 |
| Iron, mg/1,000 kcal | 0.15 | 0.18 |
| Haem iron, mg/1,000 kcal | -0.01 | 0.05 |
| Non-haem iron, mg/1,000 kcal | 0.15 | 0.17 |
| Copper, mg/1,000 kcal | 0.21 | 0.35 |
| Vitamin E, mg/1,000 kcal | 0.19 | 0.08 |
| Vitamin C, mg/1,000 kcal | 0.30 | 0.32 |
| Vitamin A, IU/1,000kcal | 0.31 | 0.43 |
| Thiamin, mg/1,000 kcal | 0.13 | 0.16 |
| Riboflavin, mg/1,000 kcal | 0.08 | 0.10 |
| Vitamin B6, mg/1,000 kcal | 0.23 | 0.32 |
| Folacin, mcg/1,000 kcal | 0.32 | 0.34 |
| Pantothenic acid, mg/1,000 kcal | 0.16 | 0.20 |
| 14-day alcohol, g/24‑h | 0.00 | -0.03 |
| Urinary sodium, mmol/24‑h | 0.01 | 0.03 |
| Urinary potassium, mmol/24‑h | 0.18 | 0.19 |
| Urinary sodium/potassium ratio | -0.13 | -0.13 |
| Urinary calcium | 0.00 | -0.01 |
| Urinary magnesium | 0.10 | 0.08 |

MFA, monounsaturated fatty acids; PFA, polyunsaturated fatty acids; SFA, saturated fatty acids; TFA, trans fatty acids.

Table S5 Gender-age-sample adjusted Pearson correlation between individual raw vegetables and individual cooked vegetables, intake per 1,000 kcal.

| Individual raw vegetables | Tomatoes | Lettuce | Onion | Garlic | Carrot | Celery | Romaine | Scallion | Cucumber | Green pepper | Ginger root |
| --- | --- | --- | --- | --- | --- | --- | --- | --- | --- | --- | --- |
| Lettuce | 0.28 |  |  |  |  |  |  |  |  |  |  |
| Onion | 0.20 | 0.05 |  |  |  |  |  |  |  |  |  |
| Garlic | 0.01 | -0.02 | 0.02 |  |  |  |  |  |  |  |  |
| Carrot | 0.10 | 0.21 | 0.00 | 0.02 |  |  |  |  |  |  |  |
| Celery | 0.04 | 0.08 | 0.03 | 0.01 | 0.17 |  |  |  |  |  |  |
| Romaine | 0.12 | 0.04 | 0.05 | 0.09 | 0.11 | 0.04 |  |  |  |  |  |
| Scallion | 0.06 | 0.08 | 0.00 | -0.02 | 0.08 | 0.22 | 0.06 |  |  |  |  |
| Cucumber | 0.18 | 0.11 | 0.05 | 0.02 | 0.04 | 0.07 | 0.08 | 0.07 |  |  |  |
| Green pepper | 0.14 | 0.16 | 0.08 | 0.01 | 0.07 | 0.13 | 0.04 | 0.08 | 0.09 |  |  |
| Ginger root | 0.00 | 0.04 | 0.13 | 0.00 | -0.01 | -0.01 | 0.01 | 0.00 | -0.02 | -0.01 |  |
| Cabbage | 0.02 | 0.08 | -0.01 | 0.01 | 0.07 | 0.06 | -0.02 | 0.16 | 0.00 | 0.14 | -0.01 |

| Individual cooked vegetables | Onion | Carrot | Green pepper | Tomato sauce | Mushroom | Celery | Canned tomatoes | Tomatoes | Green peas | Green beans | Broccoli |
| --- | --- | --- | --- | --- | --- | --- | --- | --- | --- | --- | --- |
| Carrot | 0.11 |  |  |  |  |  |  |  |  |  |  |
| Green pepper | 0.28 | 0.05 |  |  |  |  |  |  |  |  |  |
| Tomato sauce | 0.22 | -0.05 | 0.27 |  |  |  |  |  |  |  |  |
| Mushroom | 0.11 | 0.07 | 0.09 | -0.04 |  |  |  |  |  |  |  |
| Celery | 0.16 | 0.26 | 0.16 | 0.01 | 0.05 |  |  |  |  |  |  |
| Canned tomatoes | 0.28 | 0.01 | 0.24 | 0.42 | -0.01 | 0.19 |  |  |  |  |  |
| Tomatoes | 0.20 | 0.11 | 0.17 | 0.05 | 0.08 | 0.04 | -0.05 |  |  |  |  |
| Green peas | 0.05 | 0.03 | -0.02 | -0.03 | 0.01 | 0.02 | -0.03 | 0.04 |  |  |  |
| Green beans | 0.02 | 0.05 | 0.01 | -0.04 | 0.03 | 0.02 | -0.01 | 0.00 | 0.02 |  |  |
| Broccoli | -0.02 | 0.12 | 0.00 | -0.03 | 0.08 | -0.01 | -0.01 | 0.01 | -0.02 | 0.03 |  |
| Scallion | 0.04 | 0.13 | 0.10 | 0.01 | 0.08 | 0.26 | 0.04 | 0.04 | 0.03 | 0.00 | 0.03 |

Table S6. Sensitive analysis - estimated average difference in blood pressure, intakes of raw vegetables and cooked vegetables higher by 2 SD, US INTERMAP participants (N=2,195).

| ***All raw vegetables: 2 SD = 67.9 g/1,000 kcal*** | | | | | | | | | | | | | |
| --- | --- | --- | --- | --- | --- | --- | --- | --- | --- | --- | --- | --- | --- |
| Model 3 | Systolic blood pressure | | | | | |  | Diastolic blood pressure | | | | | |
|  |  |  |  | Adjusted for BMI | | |  |  |  |  | Adjusted for BMI | | |
|  | ∆BP | (95% CI) | P | ∆BP | (95% CI) | P |  | ∆BP | (95% CI) | P | ∆BP | (95% CI) | P |
| Adjusted also for total energy (kJ/day),  N=2,195 | -1.86 | (-3.02, -0.69) | 0.002 | -1.30 | (-2.42, -0.19) | 0.02 |  | -1.03 | (-1.83, -0.24) | 0.01 | -0.72 | (-1.49, 0.05) | 0.07 |
| Non-hypertensive persons,  N=1,600 | -1.35 | (-2.37, -0.33) | 0.01 | -0.90 | (-1.87, 0.07) | 0.07 |  | -0.53 | (-1.30, 0.23) | 0.2 | -0.25 | (-0.99, 0.49) | 0.5 |
| “Non-intervened” persons,  N=648 | -1.27 | (-3.27, 0.72) | 0.2 | -0.75 | (-2.69, 1.20) | 0.5 |  | -0.53 | (-1.96, 0.91) | 0.5 | -0.16 | (-1.55, 1.24) | 0.8 |
| Excluding persons with high day-to-day variability in nutrient intake and/or BP, N=1,672 | -1.65 | (-2.95, -0.34) | 0.01 | -0.87 | (-2.12, 0.39) | 0.2 |  | -0.68 | (-1.56, 0.19) | 0.1 | -0.24 | (-1.09, 0.61) | 0.6 |
| ***All cooked vegetables ^1^: 2 SD =* 92.3 g/1,000 kcal** | | | | | | | | | | | | | |
| Model 3 | Systolic blood pressure | | | | | |  | Diastolic blood pressure | | | | | |
|  |  |  |  | Adjusted for BMI | | |  |  |  |  | Adjusted for BMI | | |
|  | ∆BP | (95% CI) | P | ∆BP | (95% CI) | P |  | ∆BP | (95% CI) | P | ∆BP | (95% CI) | P |
| Adjusted also for total energy (kJ/day),  N=2,195 | -1.26 | (-2.42, -0.09) | 0.03 | -0.88 | (-1.99, 0.23) | 0.1 |  | -0.40 | (-1.20, 0.39) | 0.3 | -0.19 | (-0.96, 0.58) | 0.6 |
| Non-hypertensive persons,  N=1,600 | -1.06 | (-2.13, 0.01) | 0.05 | -0.80 | (-1.82, 0.22) | 0.1 |  | -0.58 | (-1.38, 0.22) | 0.2 | -0.42 | (-1.20, 0.35) | 0.3 |
| “Non-intervened” persons,  N=648 | -2.15 | (-4.44, 0.14) | 0.07 | -1.90 | (-4.12, 0.31) | 0.09 |  | -1.20 | (-2.84, 0.44) | 0.2 | -1.03 | (-2.62, 0.57) | 0.2 |
| Excluding persons with high day-to-day variability in nutrient intake and/or BP, N=1,672 | -0.97 | (-2.32, 0.38) | 0.2 | -0.53 | (-1.82, 0.77) | 0.4 |  | -0.39 | (-1.30, 0.51) | 0.4 | -0.15 | (-1.02, 0.73) | 0.7 |

Model 3: adjusted for age, gender, sample, year of education, physical activity during leisure time, smoking status, history of cardiovascular disease or diabetes mellitus, family history of high blood pressure, use of any special diet, use of dietary supplement, urinary sodium, 7-day alcohol, polyunsaturated fatty acids, saturated fatty acids, and cholesterol.

Table S7 Relation of raw vegetable and cooked vegetable intakes (g/1,000 kcal) to systolic and diastolic blood pressures for age, gender interaction terms and squared terms, regressed separately expressed as Z-Score, US INTERMAP participants (N=2,195).

| Model 3 | Not adjusted for  height and weight | | |  | Adjusted for  height and weight | | |
| --- | --- | --- | --- | --- | --- | --- | --- |
|  | Sex | Age | Square |  | Sex | Age | Square |
| *Raw vegetables (g/1,000 kcal)* | | | | | | | |
| Systolic blood pressure | 1.34 | 0.64 | 0.44 |  | 1.32 | -0.01 | -0.20 |
| Diastolic blood pressure | 1.40 | 0.38 | 1.24 |  | 1.38 | -0.20 | 0.72 |
|  |  |  |  |  |  |  |  |
| *Cooked vegetables (g/1,000 kcal)* | | | | | | | |
| Systolic blood pressure | 0.60 | 0.31 | 0.24 |  | 1.12 | 0.23 | -0.13 |
| Diastolic blood pressure | -0.18 | -0.21 | -0.61 |  | 0.24 | -0.30 | -0.97 |

Model 3: adjusted for age, gender, sample, year of education, physical activity during leisure time, smoking status, history of cardiovascular disease or diabetes mellitus, family history of high blood pressure, use of any special diet, use of dietary supplement, urinary sodium, 7-day alcohol, polyunsaturated fatty acids, saturated fatty acids, and cholesterol.

Table S8 Estimated average difference and 95% confidence intervals in blood pressure, intake of total vegetables higher by 2 SD, US INTERMAP participants (N=2,195).

***Sensitivity analyses***

| Model 3 | Systolic blood pressure | | | | | | |  | | Diastolic blood pressure | | | | | | |
| --- | --- | --- | --- | --- | --- | --- | --- | --- | --- | --- | --- | --- | --- | --- | --- | --- |
|  |  |  |  | Adjusted for BMI | |  |  | |  | |  |  | Adjusted for BMI | |  |  |
|  | ∆BP | (95% CI) | P | ∆BP | (95% CI) | P |  | | ∆BP | | (95% CI) | P | ∆BP | (95% CI) | P |  |
| Adjusted also for total energy (kJ/day),  N=2,195 | -2.13 | (-3.33, -0.94) | 4.8x10^-4^ | -1.50 | (-2.65, -0.35) | 0.01 |  | | -0.95 | | (-1.77, -0.13) | 0.02 | -0.58 | (-1.38, 0.22) | 0.2 |  |
| Non-hypertensive persons,  N=1,600 | -1.65 | (-2.72, -0.58) | 0.003 | -1.18 | (-2.20, -0.15) | 0.02 |  | | -0.78 | | (-1.58, 0.03) | 0.06 | -0.48 | (-1.26, 0.30) | 0.2 |  |
| “Non-intervened” persons,  N=648 | -2.41 | (-4.62, -0.20) | 0.03 | -1.87 | (-4.02, 0.27) | 0.09 |  | | -1.22 | | (-2.80, 0.37) | 0.1 | -0.84 | (-2.39, 0.70) | 0.3 |  |
| Excluding persons with high day-to-day variability in nutrient intake and/or BP, N=1,672 | -1.80 | (-3.17, -0.42) | 0.01 | -0.96 | (-2.29, 0.36) | 0.2 |  | | -0.74 | | (-1.66, 0.18) | 0.1 | -0.27 | (-1.17, 0.63) | 0.6 |  |

Model 3: adjusted for age, gender, sample, education, physical activity, smoking status, history of cardiovascular disease or diabetes mellitus, family history of high BP, use of special diet, use of dietary supplement, urinary sodium, alcohol, polyunsaturated fatty acids, saturated fatty acids, and cholesterol.
